# Selection process for botulinum toxin injections in patients with chronic-stage hemiplegic stroke: a qualitative study

**DOI:** 10.1186/s12911-019-1003-9

**Published:** 2019-12-19

**Authors:** Sawako Arai, Yuko Fukase, Akira Okii, Yoshimi Suzukamo, Toshimitsu Suga

**Affiliations:** 1 Department of Clinical Psychology, Kawasaki University of Medical Welfare, 288, Matsushima, Kurashiki, Okayama Japan; 2 Department of Health Science, School of Allied Health Sciences, Kitasato University, Sagamihara, Kanagawa Japan; 3 Physical Medicine and Rehabilitation Okii Clinic, Iwakuni, Yamaguchi Japan; 4 Tohoku University Graduate School of Medicine, Sendai, Miyagi Japan; 5 Kansai Medical University Medical Center, Moriguchi, Osaka Japan

**Keywords:** Hemiplegia, Decision-making, Botulinum toxin, Grounded theory, Spasticity, Stroke, Qualitative research

## Abstract

**Background:**

Botulinum toxin (BT) injection is a new treatment for spasticity with hemiplegia after stroke. How a patient decides to receive BT injections after becoming aware of the treatment remains unclear. In this exploratory qualitative study, we aimed to investigate patients’ decision-making about treatment strategies in collaboration with family and health professionals and to identify conflicts in patients’ feelings about BT treatment.

**Methods:**

The study included six patients with stroke sequelae. Data were collected using comprehensive interviews and were analyzed using the grounded theory approach and trajectory equifinality modeling.

**Results:**

After patients learned about BT treatment, they clearly exhibited the following two concurrent perceptions: “the restriction of one’s life due to disabilities” and “the ability to do certain things despite one’s disabilities.” Some patients reported a “fear of not being able to maintain the status quo owing to the side effects of BT.” To alleviate this fear, timely support from family members was offered, and patients overcame anxiety through creative thinking. However, there were also expressions that revealed patients’ difficulties dealing with negative events. These factors influenced the patients’ development of “expectations of BT” or “hesitations about BT.”

**Conclusions:**

To establish treatment strategies in collaboration with patients, healthcare professionals should show supportive attitudes and have discussions with patients and their family members to help patients resolve their conflicts and should establish treatment strategies that maintain the positive aspects of patients’ lives.

## Background

### Spasticity as a sequela of stroke

Spasticity is a positive symptom of upper motor neuron disorders and is commonly associated with hemiplegia after stroke [1]. It leads to various problems in a patient’s life, including difficulties with dressing, locomotion, hygiene maintenance and pain, as well as the deterioration of nutritional status [1]. These problems further aggravate spasticity, leading to a vicious cycle involving spasticity and its associated problems, such as a distorted self-image, impacts on the fulfillment of parent or partner life roles, and social isolation [1]. Additionally, these problems can result in mood disorders, loss of self-esteem, and difficulties finding a job and fulfilling social roles. This significantly affects a patient’s quality of life (QOL) [1]. When treating spasticity, setting realistic treatment goals via doctor–patient communication is important [2]. Families are recommended to participate in goal setting because treatment goals can include the relief of spasticity-associated pain and the improvement of gait, as well as the relief of family and caregiver burdens [3]. Additionally, family encouragement can help patients maintain their motivation for rehabilitation [4]. Although the importance of communication with patients, families, and healthcare providers is widely recognized, more than half of respondents in a recent survey reported that they did not receive sufficient information from their healthcare providers [5]. To treat spasticity effectively, research that contributes to improving communication between patients, families, and healthcare providers is needed.

### Botulinum toxin (BT) as a treatment for spasticity

BT injections are recommended for patients with localized or multifocal spasticity [6]. In Japan, BT injections were approved in 2010 for spasticity in the upper and lower extremities; thus, BT injections are a relatively new treatment option for treating spasticity. BT improves physical impairment by reducing muscle tone when injected into the spastic muscles [7] and improves behavioral and psychological problems, such as those that impact QOL, by increasing activity levels, e.g., by improving limb posture and ways of dressing, which are easily disturbed by spasticity [8]. However, some studies have reported that no increase in activity or improvement in QOL was observed after BT injections, even though spasticity decreased [9]. Thus, the effects of BT treatment remain controversial.

### How does a patient select the treatment for spasticity for the first time?

There is a global shift from decision-making by healthcare providers to health-related decision-making by patients. In a large-scale survey on health decisions in Canada [10], 59% of respondents reported that they had experienced decision-related conflicts and that they often shared the process with a partner/spouse (63%) or another family member (27%). The necessity of support from reliable information sources and appropriate information to aid decision-making has been mentioned [10, 11]. In addition, help with decision-making may have a positive influence on general health and physical function [12]. Reportedly, healthcare professionals have provided services to patients based on trial and error because research on BT treatment for spasticity as a stroke sequela at the chronic stage is in the preliminary phase of data collection [13]. A survey [14] of six patients with spasmodic dysphonia who received BT for a long period revealed that BT has multifaceted psychosocial implications in the physical, personal, and social realms and that early experience with BT plays an important role in predicting the outcomes of subsequent treatment. Before receiving the first BT injection, a patient experiences conflict between the fear of injecting the poison into his/her throat and the additional effort, fatigue, and occupational difficulties imposed by spasticity, which exhaust the patient on a daily basis. However, the process involved in a patient’s decision to receive BT has not been clarified.

It remains unknown how healthcare professionals and a patient’s family members interact with the patient before and after BT treatment, whether the patient accepts interventions by healthcare professionals and family members and how he/she thinks about and acts in response to these interventions. We believe that it is important to understand how a patient makes the decision to receive a BT injection after learning about it as a treatment for spasticity and after considering all other approaches suggested by healthcare professionals and family members. Understanding the process is necessary to build such treatment strategies for those receiving BT treatment.

### Importance of qualitative studies

Qualitative methods have been used to examine the experience of disability in patients with chronic illnesses, including stroke patients with hemiplegia at the chronic stage. Qualitative research is effective in that it not only informs the establishment of new treatment models but also allows close observation of events, identifies certain patterns, and reveals a phenomenon through relevant concepts. The grounded theory approach (GTA) [15] is a typical qualitative method that allows the systematic consolidation of qualitative data into new models. However, past models based on the GTA had several shortcomings; e.g., they aimed to create models of disability acceptance, and the findings rarely supported treatment selection and improvement in QOL. Moreover, they primarily evaluated aspects of a patient’s psychological state, including dysphoria and anguish, but not behavioral outcomes of the interactions between a patient and his/her environment.

Therefore, in this study, we used trajectory equifinality modeling (TEM) [16] in addition to the GTA. TEM is a new qualitative approach that depicts individual experiences on a timeline, thus making it possible to describe an individual’s activities and the phases of decision-making in the context of environmental influences. TEM can be combined with the GTA [17]. We believed that a qualitative study that combined TEM and the GTA could reveal the diverse experiences of stroke patients during the process of treatment selection and the influence of family members and healthcare professionals on patients’ decision-making processes.

### Aims

The primary aim of this study was to investigate the decision-making of patients with stroke who had considered BT treatment by examining their interactions with family members and healthcare professionals. Additionally, the study aimed to identify the conflicts in patients’ feelings about BT treatment for stroke. Based on the results, we discuss how healthcare professionals should approach patients and their family members to assist with treatment decision-making processes.

## Methods

### Participants

The study included six patients with stroke sequelae who were outpatients at three Japanese hospitals and were recommended BT injections between May 2014 and September 2016. Table 1 summarizes the inclusion criteria.

**Table 1:** Study inclusion and exclusion criteria

Inclusion criteria	Exclusion criteria
1 Patients who experienced stroke at least 6 months prior to the study period	1 Patients who had previously received BT injections
2 Patients who had spasticity as a stroke sequela	2 Patients with aphasia
3 Patients who lived at home	3 Patients who experienced stroke recurrence within 1 year prior to the beginning of the study
4 Patients who planned to receive a BT injection for the first time	4 Patients with a history of other central nervous system disorders
5 Patients aged 20–85 years	5 Patients who had severe internal impairments that could decrease exercise tolerance
6 Males and females	6 Patients with suspected dementia (who scored less than 23 on the Mini-Mental State Examination (MMSE) or scored less than 20 on the revised Hasegawa’s Dementia Scale (HDS-R))
7 Patients who could have a daily conversation	7 Patients with depression (with scores of more than 11 on the depression subscale of the hospital anxiety and depression scale (HAD), a score of less than one standard deviation below the mental health subscale of the 36-Item Short Form (SF-36) or a score of more than 7 on the 30-item General Health Questionnaire (GHQ30))
8 Patients who clearly understood the process and provided written consent after receiving adequate information about the study	8 Patients who were assessed as unsuitable by the physician in charge of participant recruitment

The general descriptions of patients’ physical functions prior to the injections are described in Tables 2 and 3.

**Table 2:** Patient descriptions

ID	Age	MMSE or HDS-R score	Onset (years prior to the study)	Functional autonomy	How did the patient learn about BT and make a decision?
A	45–49	MMSE = 24	5	Could go out with a cane and a lower extremity orthosis.	BT was introduced by the primary physician.
					Patient selected BT injection to enhance gait stability and improve upper limb activity.
B	55–59	MMSE = 29	10	Could go out with a cane and a lower extremity orthosis.	BT was introduced by the primary physician.
					Patient selected BT injection to enhance gait stability and improve upper limb activity.
C	60–64	HDS-R = 26	4	Could go out.	BT recommended for a tingling sensation in the foot.
					Patient did not select BT injection owing to the possibility of convulsions.
D	60–64	MMSE = 28	8	Could go out with a cane and a lower extremity orthosis under supervision.	Patient was looking for better treatment options and obtained information about BT.
					Selected BT injection to enhance gait stability and improve upper limb activity.
E	60–64	HDS-R = 26	9	Could go out with a cane and a lower extremity orthosis.	Patient heard about BT from patients who had received BT and healthcare professionals. Selected BT injection to enhance gait stability.
F	70–74	MMSE = 26	6	Could go out with a cane and a lower extremity orthosis under supervision.	BT recommended by healthcare professionals and family members.
					Selected BT injection to enhance gait stability and improve upper limb activity.

*BT* Botulinum toxin, *MMSE* Mini-Mental State Examination, *HDS-R* Revised Hasegawa’s Dementia Scale

**Table 3:** Physical function of the patients (prior to the BT injection)

ID	BRS			MAS			DKE	LSA
	Upper limb	Lower limb	Hand	Leg	Elbow	Wrist		
A	V	IV	IV	3	1+	2	0	82.5
B	III	IV	II	1+	1+	2	5	67
C	III	III	III	2	2	2	0	39
D	III	II	III	2	3	3	−8	39
E	III	II	III	3	4	4	−40	54
F	III	II	IV	2	2	1+	−5	34

*BRS* Brunnstrom recovery stage, *MAS* Modified Ashworth Scale, *DKE* Dorsiflexion with knee extension, *LSA* Life-Space Assessment

### Survey methods and items

A semistructured interview (lasting 1 h on average) was conducted using an interview questionnaire. The interview was scheduled between when the patients received an explanation about BT treatment from a physician and when the patients received the first injection or rejected the injection. A physician explained the prognosis of stroke and spasticity, treatments for spasticity, and the active mechanisms associated with BT treatment. The provision of information was semistructured. Furthermore, the physician explained the BT injection site, the effects and side effects of BT injection, what to expect after a BT injection, the cost of BT injections, the method of BT injection, the time required for BT injections, and rehabilitation after receiving the injection. This process generally took approximately 30 min. After explaining these details, the physician asked the patient if there were any questions and answered the patient’s queries until all were resolved. The physician also distributed a booklet and a document about stroke and its sequelae, the treatment of spasticity and BT treatment, how to proceed with BT treatment, the effects of BT, the side effects of BT, and precautions for after BT injection (see Additional files 1 and 2). Thus, the physician did not immediately give BT injections on the patients’ first visits but gave them and their families more than a week to think about the treatment.

The interview questionnaire was developed based on a pilot study and discussions with a physician and two clinical psychologists. The pilot study used a preinterview questionnaire that was created using selected research questions about how healthcare professionals and family members approach a patient before and after BT treatment, whether the patient accepts interventions by healthcare professionals and family members and how he/she thinks about and acts in response to these interventions.

In the pilot study, an interview centered around the themes of “the circumstances of learning about BT and the period after learning about BT” and “understanding and thinking about treatment after the first examination” was conducted with one 55–64-year-old patient. The interview items were created based on the research questions (how healthcare professionals and family members approach a patient before and after BT treatment, whether the patient accepts interventions by healthcare professionals and family members and how he/she thinks and acts in response to these interventions).

The interviews revealed that patients addressed inconveniences caused by spasticity by using various treatments, services, and self-help efforts before considering treatment for BT. We found that these experiences were closely related to the decision-making process for BT treatment. Therefore, after the pilot study, the physician and the clinical psychologists discussed and added two interview items ([Daily life after the onset and evaluation of symptoms] and [Treatment and rehabilitation received in the past]) to understand past experiences.

Then, the clinician and the clinical psychologists modified wording, arranged the items in a time series sequence, and added examples. These modified questionnaire items were used to create the interview guide (Table 4). Information about which patients received the injection was obtained from the physician who injected the patients after the interview.

**Table 4:** Interview guide

[Daily life after the onset and evaluation of symptoms]	
- Please tell me about your physical condition after experiencing stroke in terms of your daily life; e.g., how do you spend your day?	
[Treatment and rehabilitation received in the past]	
- Please tell me in chronological order the types of treatments and rehabilitation approaches have you tried.	
- After discharge, many services, such as long-term care insurance, are available; what types of services are you currently using?	
- Please tell me about the adjustments you have made and how you have handled daily matters since returning home.	
[The circumstances of your introduction to BT and the period afterwards]	
- How did you obtain information about BT? What were your thoughts about it afterwards?	
- What did your family say about the topic of BT?	
- Have you ever heard about someone who has been treated with BT?	
- What happened before you scheduled an appointment for a BT injection?	
- Did you collect any information about BT?	
[Understanding and thinking about treatment after the first examination]	
- What did you think after you met the physician (who was supposed to give the BT injection) and received an explanation about the injection?	
• What were your hopes or expectations regarding the BT injection?	

To evaluate the physical function of the patients, the following four indices were measured during the interview: 1) the Brunnstrom recovery stage [18], which is one of six stages (I–VI), with a lower stage indicating a higher degree of paralysis; 2) the Modified Ashworth Scale [19], comprising six stages (0–4), with a lower stage indicating less spasticity; 3) dorsiflexion with knee extension [20], which indicates the range of motion of ankle dorsiflexion with the knee extended, with a lower score indicating a lesser degree of equinus foot; and 4) the Life-Space Assessment [21, 22], with a score of 0 to 120 points, with a higher score indicating a wider range of activities.

### Methods of analysis

The interviews were transcribed verbatim, and a qualitative analysis was performed. TEM and the GTA were used to analyze the events leading up to treatment selection [16, 23]. The GTA was developed by Saiki-Craighill using the method published by Corbin and Strauss in 2008 to analyze psychological experiences [23].

TEM is a qualitative method that is used to analyze various behaviors, choices, and changes in awareness along a timeline. It describes external forces such as cultural and social influences on the behavioral change process to identify the bifurcation point and the equifinality point that many people reach. It illustrates the paths of behavioral change created because of the interactions of various forces. External forces are categorized as social guidance (SG), indicating that a force has the tendency to push toward an equifinality point, and social direction (SD), indicating that a force does not push toward an equifinality point.

The GTA is a method of qualitative analysis that is used to create a theory by adhering to qualitative data. Precisely classified data are coded according to the characteristics of the data, and categories (concepts) and themes are extracted following the coding of data. Through linking categories in an orderly sequence, theories are derived from phenomena.

When coding the data according to the GTA, the narratives related to treatment selection after the bifurcation point identified by TEM were extracted and divided into the smallest coherent episodes. The episodes were coded to clarify their characteristics. In addition, a label name appropriate for the semantic content or theme of each episode was assigned based on the code. Similar labels were collected into a group and assigned a category name.

The analysis was conducted by two researchers, who were not the physicians who administered the BT injections. First, using TEM, the onset of stroke was set as the starting point, and the point when the patient selected or did not select BT treatment was set as the equifinality point. Patient experiences were arranged in chronological order between the starting and equifinality points, and the external forces that affected treatment selection, according to family members and healthcare professionals, were identified as SD or SG. Then, a turning point was identified from the patients’ experiences that had significantly influenced treatment selection. This turning point was set as the bifurcation point. The patients’ inner experiences that occurred after the bifurcation point were comprehensively analyzed according to the GTA. For the GTA, patients were individually analyzed, and then a comparative analysis was conducted. Finally, an association chart that linked categories with other extracted categories was developed.

## Results

**Fig. 1:**
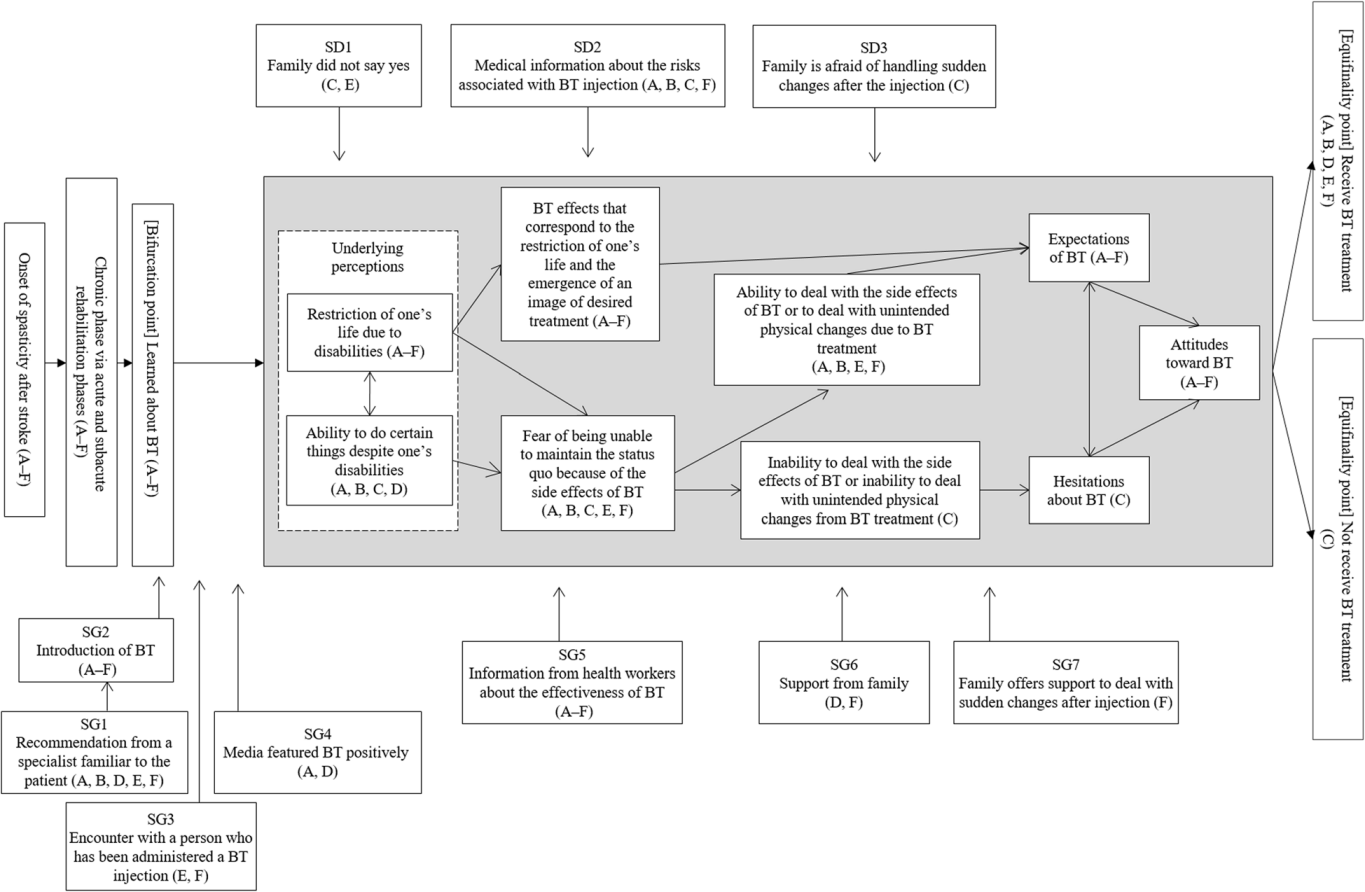
TEM and association charts of the categories identified through the GTA. The gray area shows the results of the GTA, and the other areas show the TEM results. In the trajectory equifinality model, the right arrows indicate the processes of the patients, the downward arrows indicate forces that did not have the tendency to push toward an equifinality point (social direction), and the upward arrows indicate forces that did have the tendency to push toward an equifinality point (social guidance). GTA, grounded theory approach; TEM, trajectory equifinality modeling

Figure 1 shows the trajectory equifinality model and the association chart of the categories identified through the GTA (shaded part of Fig. 1), which was developed through qualitative analysis. Each category is marked with the IDs of patients who had experiences that corresponded to the category. In the text, when describing the trajectory equifinality model and the category association charts, both the TEM and grounded theory categories are indicated in quotation marks, and examples of patients’ remarks are shown in quotation marks and italics. The portions of the quotations in parentheses are participants’ inferred remarks during the interviews.

### TEM results: influences of family members and healthcare professionals, with a central focus on when patients “learned about BT”

The following explanation concerns TEM of patients’ experiences from the onset of stroke to BT selection. The path to “receive BT treatment” or “not receive BT treatment” begins from the “onset of spasticity after stroke” and continues through the “chronic phase via acute and subacute rehabilitation phases”; the bifurcation point that indicates when patients “learned about BT.”

SD and SG were located between the bifurcation point and treatment selection in the trajectory equifinality model. SD refers to external forces that cause a patient to hesitate to accept BT treatment; the categories of SD were various external influences from family members, such as “the family did not say yes” [SD1], e.g., “(*I urged my partner to go take an injection, but) she was indecisive about letting me undergo the treatment*”; “the family is afraid of handling sudden changes after the injection” [SD3], e.g., “*(My partner is) blind, and because she cannot use the phone, she cannot call an ambulance*”; and “medical information about the risks associated with BT injection” [SD2], e.g., “*The doctor told me several times in advance that there are such side effects*.” On the other hand, SG refers to external forces that cause patients to accept BT treatment; one category of SG was “a recommendation from a specialist familiar to the patient” [SG1], e.g., “*(The therapist in the rehabilitation center) found a clinic (that administers BT injection) on the Internet. He gave me the address and telephone number of the clinic in writing*” and “*(The visiting rehabilitation therapist) recommended this therapy. He presented at a seminar and asked me, “How about this treatment?*” In addition, the following categories of SG that influenced patients at about the same point were identified: “the introduction of BT” [SG2], e.g., “*During consultation for the upper limbs, he recommended such treatments*”; “an encounter with a person who has been administered a BT injection” [SG3], e.g., “*An acquaintance of mine whose hands were extremely rigid is receiving treatment from the doctor. Seeing her progress, I am hopeful that even my hands will get better*”; and “the media featured BT positively” [SG4], e.g., “*I have watched BT-related programs on the Internet several times. (With this treatment), I believe that the condition may improve wonderfully.*” The other categories of SG included “information from healthcare workers about the effectiveness of BT” [SG5], e.g., “*It was explained that the muscles will become soft*”; “support from family” [SG6], e.g., “*My partner said that I may recover (by this treatment), and she contacted the therapist immediately*”; and “the family offers support to deal with sudden changes after injection” [SG7], e.g., “*My daughter suggested that she will come home for about a week to administer this injection (probably because someone felt slightly dizzy after it)*.”

Both “medical information about the risks associated with BT injection” [SD2] and “information from healthcare workers about the effectiveness of BT” [SG5] were related to information about BT injections from healthcare professionals and descriptive pamphlets.

Eventually, five patients decided to “receive BT treatment,” and only one patient (patient C) decided to “not receive BT treatment.”

### Results of the GTA: patients’ conflicts from the time they “learned about BT” to “treatment selection”

When asked about their personal experiences, the patients reported the following two concurrent perceptions: 1) “the restriction of one’s life due to disabilities,” e.g., “*I cannot live a normal life. I cannot use my right hand, and there is the inconvenience of immediately falling if I do not wear a brace on my right foot,*” and 2) “the ability to do certain things despite one’s disabilities,” e.g., “*Grab a towel with the right hand and squeeze it with the left hand. Squeeze it with both hands. I can squeeze for now.*” By understanding BT, the patients began to shift from a focus on “the restriction of one’s life due to disabilities” to a focus on the “BT effects that correspond to the restriction of one’s life and the emergence of an image of desired treatment,” e.g., “*If I receive this injection, I believe that my legs will turn a little toward the outside.*” Thus, the patients formed “expectations of BT,” e.g., “*It seemed like a dream injection.*” On the other hand, while listening to various details about BT, participants received medical information about the risks. Additionally, external forces were identified based on participants’ beliefs in “the ability to do certain things despite one’s disabilities” shifting to a “fear of being unable to maintain the status quo because of the side effects of BT,” e.g., “*I can hold a PET bottle even with spasticity*; *I will have trouble if BT induces too much relaxation*.” Most patients shifted from having a “fear of being unable to maintain the status quo because of the side effects of BT” to believing in their “ability to deal with the side effects of BT or to deal with unintended physical changes due to BT treatment,” e.g., “*I think I will inject a small amount*” and “*If I get concerned, I will not receive the treatment, (so I will not mind)*.” However, patient C shifted from having a “fear of being unable to maintain the status quo because of the side effects of BT” to perceiving an “inability to deal with the side effects of BT or inability to deal with unintended physical changes from BT treatment,” e.g., “*Convulsions are the most terrible thing. They are painful. My partner created a fuss when I experienced convulsions in the past. It is serious if convulsions occur at home. I fear it*.” Additionally, this patient noted, “*I will not receive the injections (if there is a possibility of occurrence of side effects)*”; thus, there was a conflict between the patient’s “expectations of BT” and “hesitations about BT.”

Patient F did not clearly express feelings about “the ability to do certain things despite one’s disabilities”; nevertheless, after obtaining medical information, this patient developed a “fear of being unable to maintain the status quo because of the side effects of BT,” e.g., “*I heard that it is better to be careful (after the therapy). Someone probably felt slightly dizzy after it*.” .

### Interactions between patients and others: relationship between patients’ inner experiences and external forces identified via TEM and the GTA

In the trajectory equifinality model, at the bifurcation point “learned about BT,” external forces that directed the selection of injection [SG] were the primary factor. However, the diversity of sources of information about BT treatment led to various perceptions and diverse expectations as well as hesitations, e.g., “*I think I’ll be saved … I have been in a tunnel, and I was in pitch darkness. I finally see the light at the end of the tunnel*” and “*I went up to speak (to the person who had BT). … (The person) was telling the physical therapist that the pain had decreased quite a lot. I wish it would work on me too. (I think) it would be great if the pain in my foot decreased a bit*.”

As shown in the association chart of the categories identified through the GTA, patients’ recognition of “BT effects that correspond to the restriction of one’s life and the emergence of an image of desired treatment” developed when information was provided by healthcare professionals [SG5], and the BT effects that were explained by healthcare professionals corresponded to patients’ underlying perceptions of “the restriction of one’s life due to disabilities” that they had originally expressed. Thereafter, various “expectations of BT” were formed, e.g., “*(The condition improves wonderfully, I think). That was my pleasure*”; “*I hope it relieves my foot pain at least a little*”; “*I would like to walk easily*”; and “*I would like to get a little better (about keyboard input skills).*” The “fear of being unable to maintain the status quo because of the side effects of BT” also developed in some patients who received medical information from healthcare professionals [SD2]. Timely support offered by family members [SG6 and 7], and patients’ own creative thinking alleviated such fear, resulting in perceptions such as the following: “*I am afraid of what would happen to me if a complete injection was performed the first time. So, I think I can deal with the treatment if small amounts are injected.*” However, there were expressions of fear from family members [SD3] and feelings that the patient could not deal with negative events, such as “*(I am) afraid of convulsions. I cannot deal with it (if convulsions happen to me).*” These factors influenced patients’ development of either expectations of BT or hesitations about BT.

Although patient C was apprehensive about BT, this patient developed “expectations of BT,” stating “*I want to fix the tingling feeling in my foot*.” Both “expectations of BT” and “hesitations about BT” coexisted in this patient, and the patient developed “attitudes toward BT” that combined both feelings.

## Discussion

### Providing treatment information

The results of this study suggested that the patients obtained different details about BT from multiple sources and vacillated between acceptance and refusal of treatment due to difficulties in their daily lives as well as their fears of losing bodily functions. When they again received medical information on BT, they developed either positive (“expectations of BT”) or negative (“hesitations about BT”) attitudes about BT, and they were also influenced by their family members.

A study by therapists in Australia [13] demonstrated that patients receiving BT were involved with multiple healthcare professionals who experienced difficulties cooperating with each other. Furthermore, the study reported that these problems were associated with healthcare professionals’ lack of clarity about the type of information that should be provided to a patient. In addition, it has been suggested that information is an important aid to decision-making [10, 11]. The present study showed that patients had already experienced various encounters with BT before healthcare professionals provided them with information.

The findings of this study suggested that the diverse sources of information on BT influenced patients’ subsequent perceptions of the treatment. Caty et al. [24] indicated the gap between the effects of one BT injection and patients’ expectations to be one reason why BT injections did not lead to improved participation in the International Classification of Functioning, Disability and Health [25] or improvements in QOL. To narrow this gap, it is important for healthcare professionals to understand how a patient perceives BT, what information the patient already has before they provide information, and what a patient expects from the treatment.

In the current study, in addition to information about the effects of a BT injection, information about risks provided by healthcare professionals seemed to influence patients’ and family members’ participation in treatment by increasing their efforts to allay their concerns. Our study suggested that the concerns and support of family members influenced the development of patients’ “attitudes toward BT.”

Previous studies have indicated the importance of healthcare professionals’ cooperation with a patient’s family [2] because treatment goals often include reducing caregiver burden and encouraging the patient to engage in active rehabilitation. This study suggested that both family cooperation and unease significantly influenced patients’ motivation to engage in treatment.

On the basis of these findings, we highlighted the following as noteworthy considerations when healthcare professionals share information with patients: 1) provide accurate information about treatment benefits and harms; 2) actively note concerns and inquiries and discuss them with the patient (e.g., provide advice about the injection site and dosage control to patients who have concerns about risks); and 3) ensure that points 1 and 2 are also achieved with family members. (It is desirable that the patient and his/her family members are all present when information is provided.)

Notably, shared decision-making is more important when there are multiple options or when patient preferences and values are strongly influenced by the selection than when the evidence strongly supports a single clearly superior option [26].

Since there are multiple options for the treatment of spasticity, shared decision-making in BT treatment is important. Such strategies are common to guide the process of sharing decision-making. It has been noted that there are barriers that prevent the adoption of sharing decision-making [26], and the use of such consent forms and booklets is considered useful to broaden engagement in sharing decision-making.

### Providing support for treatment selection based on a patient’s inner experiences

As shown in the grounded theory category association chart, stroke patients with hemiplegia at the chronic phase had pre-existing conditions that they felt restricted their daily lives. In addition, they regained some abilities via rehabilitation and their efforts after the onset of stroke and were afraid of losing their abilities after receiving BT treatment. Their families supported seeking BT treatment but, at the same time, said that they were afraid of the risks associated with BT injection. Due to their interactions with their families, patients experienced conflict between the “expectations of BT” and “hesitations about BT,” and these conflicts developed their “attitudes toward BT.”

Japanese individuals have stronger desires for information about treatments and prognoses for stroke and paralysis than for other diseases, such as terminal cancer, for which a good treatment prognosis cannot be expected [27]. In addition, they tend to desire to consult with and entrust judgment to healthcare professionals and family members and avoid individual decision-making when they need to make decisions about medical care, such as treatment methods and lifestyles [28]. For example, conflicts arose in decision-making and treatment selection for patients with breast cancer who were told to individually select operative methods. They experienced conflicts between the hope for breast conservation and the fear of cancer recurrence and postoperative radiation therapy; nevertheless, this process facilitated a realistic and concrete thought process regarding operative method selection [29]. Patients hospitalized with a lack of awareness of appropriate decision-making can become confused and swayed by excess information [29]. In a similar vein, for patients seeking BT treatment, appropriate and early support is required to determine treatment selection. Among patients in the current study, conflicts about treatment selection played an important role during decision-making. To support patients’ choices, it is important for healthcare professionals to acknowledge patients’ conflicts and show supportive attitudes toward patients’ conflicts to prevent any confusion after treatment.

In treatment for spasticity, it is important to educate patients and caregivers carefully before initiating treatment and to discuss realistic treatment goals, including how much improvement should be expected in daily life difficulties associated with spasticity, such as difficulties in dressing, pain, and decreased locomotion [1, 30]. A systematic review of neurorehabilitation outcomes after BT reported that a mechanism for capturing how benefits can be applied to daily life is needed [31]. The characteristics of the conflicts observed in our study were based on the patients’ everyday lives, including conflicts between perceptions of “BT effects that correspond to the restriction of one’s life and the emergence of an image of desired treatment” and the “fear of being unable to maintain the status quo because of the side effects of BT.” Addressing these conflicts requires thinking about how to apply treatment benefits to a patient’s daily life [31]. In response to these conflicts, healthcare professionals should take the following steps to provide support prior to treatment to enhance the development of patients’ decision-making processes: 1) verify the patient’s current condition (i.e., the lifestyle that a patient is currently able to lead); 2) provide information on risk management as well as on the benefits of the patient’s lifestyle; and 3) establish treatment strategies that maintain the positive aspects of the patient’s present lifestyle through discussions with the patient and his/her family members.

### Efficacy of the combined study method involving TEM and the GTA

From the various existing qualitative methods, we chose to use TEM and the GTA to create a BT treatment support model. The findings indicated that some factors appeared to influence patients’ decision-making, as described below. Future quantitative research is required to verify this hypothesis.

Using TEM, which follows a timeline (a chronological order of events), we were able to visualize the sequence of events that occurred when healthcare professionals offered support to patients in chronological order. Through the description of phenomena according to external forces, which is characteristic of TEM, a variety of individual perspectives that developed during the process of treatment selection were identified. In TEM, a variety of experiences can be depicted when the number of interviewed patients is 4 ± 1 [32]; thus, in our study, a variety of patient experiences were described.

The patients were influenced by family members and healthcare professionals, and they developed expectations and fears throughout these experiences. The application of the GTA to create an internal model was valid for understanding psychological conflicts and decision-making based on patients’ interactions with others. The analysis combining TEM and the GTA suggested that the sources and content of information about treatment were important for supporting patients who followed a variety of decision-making pathways.

### Limitations

Our results are not generalizable to other populations. There were six participants in this study; therefore, it seems that an analysis of the interactions between people who wanted treatment and the surroundings was possible in the TEM analysis. However, theoretical saturation was not reached in the grounded theory analysis. To achieve theoretical saturation, it is necessary to collect data until new categories and themes are no longer being generated. In particular, the categories for conflicts regarding treatment were not sufficient; therefore, further research with participants who have not decided whether to have BT treatment is needed.

Additionally, although we recruited patients from three facilities, only six patients participated, and only one decided not to undergo BT treatment. Thus, we were unable to adequately analyze patients who did not select BT treatment.

Since the remaining participants were only those who were planning to undergo BT treatment, we could not provide a balanced perspective on decision-making about BT treatment. To achieve such a balance, participants who had decided to not receive a BT injection after being informed about it should also have been recruited and interviewed.

To support patients’ decision-making, it will be necessary to include multiple patients who opt out of BT treatment and to consider the differences in their living conditions and disability levels, especially for patients do not select treatment despite family support. Some patients have positive feelings toward the treatment but do not select the treatment owing to family members’ concerns. In addition, follow-up studies after BT injections will be required to assess how differences in patient decision-making process are associated with the degree of satisfaction, an increase in the range of activities, and the subsequent continuation of treatment.

Although participants in this study were not considered to have dementia according to their MMSE and HDS-R scores, and although physicians concluded that all participants possessed sufficient competency to provide informed consent according to the four elements of decisional capacity outlined by Appelbaum and Grisso [33], it is possible to have an MMSE score greater than 20 and be unable to adequately comprehend informed consent [34]. The addition of neuropsychological tests to measure different cognitive functions would clarify the influence of cognitive function on decision-making in this population.

## Conclusion

To establish treatment strategies to facilitate and improve patients’ treatment selections, healthcare professionals should show supportive attitudes when addressing conflicts and should build treatment strategies that maintain patients’ present lifestyles through discussions with patients and their family members.

## Supplementary information

**Additional file 1.** Consent form. The consent form was provided by a pharmaceutical company to be distributed to patients. We confirmed with the pharmaceutical company that created the agreement form that they were only available in Japanese. Therefore, we translated the consent form from Japanese into English.

**Additional file 2.** Table of contents of the booklet. The booklet was provided by a pharmaceutical company to be distributed to patients. We confirmed with the pharmaceutical company that created the booklet that they were only available in Japanese. Therefore, we translated the table of contents of the booklet from Japanese into English.

## Data Availability

The datasets analyzed during the current study are available from the corresponding author on reasonable request.

## Abbreviations

BT: Botulinum toxin
GTA: Grounded theory approach
LSA: Life-Space Assessment
QOL: Quality of life
SD: Social direction
SG: Social guidance
TEM: Trajectory equifinality modeling
