# Supplementary material for: Selection process for botulinum toxin injections in patients with chronic-stage hemiplegic stroke: a qualitative study
Source: BMC Med Inform Decis Mak. 2019 Dec 19;19:280. doi: 10.1186/s12911-019-1003-9 (PMC6923967; doi:10.1186/s12911-019-1003-9)
Supplement: Supplementary file 1 — Additional file 1. Consent form. The consent form was provided by a pharmaceutical company to be distributed to patients. We confirmed with the pharmaceutical company that created the agreement form that they were only available in Japanese. Therefore, we translated the consent form from Japanese into English. [file 12911_2019_1003_MOESM1_ESM.pdf]

Botox Consent Form

For spasmodic torticollis, arm spasm, leg spasm and cerebral palsy

[Manufacturer] (Importer)

GlaxoSmithKline K.K.

Questions regarding Botox/Information request

Phone: 0120-561-703 (9:00-17:45/except Saturdays, Sundays, public holidays and company holidays)

Fax: 0120-561-047 (24-hour line)

Revised: February 2019

(P3) For patients

For patients with spasmodic torticollis, arm spasm, leg spasm and cerebral palsy

Treatment with Botox

1. Your condition
The condition you suffer from is called "spasmodic torticollis," "arm spasm," "leg spasm," or "talipes equinus caused by leg spasm in pediatric cerebral palsy." "Spasmodic torticollis" is abnormal tension of the muscles surrounding the neck, leading to an unnatural posture of the head, neck and shoulders. It can also present as severe chronic pain in the shoulders and neck with a normal posture. "Arm spasm" and "leg spasm" are types of motor dysfunction caused by a stroke or neurological disease and are characterized by excessive muscle tension, which leads to difficult or involuntary movement of the arm and leg. Some of the common symptoms include difficulty opening the hand with the fingers trapped in a fist, difficulty bending the elbow, and toes pointing towards the sole of the foot. "Talipes equinus caused by leg spasm in pediatric cerebral palsy" is caused by cerebral palsy, which results in abnormal tension of the leg muscles and inability to place the heel on the ground.<br><br>The medication we recommend to you here is an injectable medicine called Botox. Botox has been approved in more than 90 countries worldwide, including the US and UK (as of January 2019). You need to fully understand and agree to the following points before receiving treatment with this medication. If you have any questions or concerns about this treatment, please do not hesitate to ask.
**2. Active ingredient**
The active ingredient in this medication is a natural protein called botulinum toxin type A, which is produced by bacteria called *Clostridium botulinum*. The bacteria itself are not injected, so there is no risk of becoming infected with *Clostridium botulinum*. Various studies have shown that direct injection of a very small amount of this protein into tense muscles leads to their relaxation, producing relief from muscle tension and convulsions. This active ingredient is used in pharmaceutical products.
**3. Effects**
◆ The effects of this medication will appear 2-3 days to 2 weeks after injection and will typically continue for 3-4 months. With time, the effects will gradually disappear and nerve function will recover. Because of this, your pre-injection condition will appear again. In this situation, re-injection of Botox will produce similar effects.
◆ Because protein is the main ingredient of this medication, your body might, although very rarely, produce antibodies with the continuation of treatment, thereby diminishing the effects.
**4. Side effects**
◆ This medication was given to 10,645 patients with spasmodic torticollis. Side effects were reported in 508 patients (4.77%), including difficulty in swallowing (dysphagia) in 208 patients (1.95%), localized muscle weakness in 89 patients (0.84%), and feeling of weakness in 31 patients (0.29%).
◆ One case of sudden death was reported in a Japanese domestic clinical trial of spasmodic torticollis. The attending physician ruled out a medical causal relationship with this medication. However, due to insufficient data, the cause could not be determined and a causal relationship with this medication could not be completely ruled out.
◆ A major Japanese domestic clinical trial including a total of 106 patients presenting arm spasm after a stroke reported side effects after Botox injection, including abnormal laboratory tests in 17 patients (16.04%). The main side effects included weakness in three patients (2.83%) and increased creatine kinase (CPK) levels in three patients (2.83%).
◆ A major Japanese domestic clinical trial including a total of 115 patients presenting leg spasm after a stroke reported side effects, including abnormal laboratory tests in 18 patients (15.65%). The main side effects included injection site pain in five patients (4.35%), muscle pain in three patients (2.61%), and rash in two patients (1.74%).
◆ Post-market surveillance of Botox use in patients with arm spasm and leg spasm reported side effects in 18 out of 995 patients (1.81%). The main side effects included muscle weakness in three patients (0.30%), double vision in two patients (0.20%), and injection site pain in two patients (0.20%).
◆ An overseas clinical trial that included 215 ≥2-year-old pediatric cerebral palsy patients with leg spasms presenting talipes equinus reported side effects of Botox in 67 patients (31%). The main side effects included falling in 20 patients (9%), leg pain in five patients (2%), leg weakness in five patients (2%), and whole-body weakness in four patients (2%).
◆ Most of these side effects were considered a manifestation of stronger than expected drug action. Thus, the side effects recovered with weakening of the drug effect.

**** Please contact a physician if you experience the following symptoms:**

Potential allergic side effects, including skin symptoms (such as rash and itchiness), gastrointestinal symptoms (such as nausea and abdominal pain), respiratory symptoms (such as breathlessness and hoarseness), and shock symptoms (such as clouding of consciousness). These symptoms might be caused by anaphylaxis (severe allergic response) or serum sickness.

After an injection into the muscle at the front of your neck, you might experience side effects such as difficulty in swallowing food, changes in your voice, or difficulty breathing. This occurs due to changes in the tension or coordination of the neck and throat muscles. Typically, mild symptoms will recover in a few weeks. However, since there is an overseas report of a death caused by aspiration pneumonia from the accidental inhalation of food in an elderly patient who received Botox, please contact a physician immediately if such symptoms appear.

After treatment, you might experience convulsions. This is known to occur more frequently in patients with a history of convulsions. Please contact a physician if you experience such symptoms.

The effects of this medication will typically last for 3-4 months. Side effects not listed here might also appear. If you become ill while being treated with this medication, or if you have any concerns about treatment with this medication, please contact your doctor.

If you experience changes in your physical condition, such as development of breathlessness or weakness, during the 3-4-month period after injection of this medication, please contact your doctor immediately.

5. Other precautions

- ◆ Patients with systemic muscle weakness (such as myasthenia gravis, Lambert-Eaton myasthenic syndrome and amyotrophic lateral sclerosis) cannot use this medication as it might worsen these conditions.
- ◆ Its safety in fetuses and infants has not been established. Women who are pregnant or nursing cannot use this medication. Women who are planning on becoming pregnant must use acceptable forms of birth control during Botox treatment and for two menstrual cycles after the final Botox treatment.
- ◆ Men must use acceptable forms of birth control during Botox treatment and for at least 3 months after the final Botox treatment.
- ◆ Please notify your doctor if you have previously used this medication and experienced an allergic reaction such as a rash, or if you have allergic tendencies.
- ◆ Please notify your doctor of all medications you are taking, including over-the-counter drugs. In particular, if you are taking aminoglycoside antibiotics, medication for Parkinson’s disease, muscle relaxants, tranquilizers, or were treated with another botulinum toxin, notify your doctor and follow instructions. This is important because the simultaneous use of Botox may result in a stronger effect of Botox and requires careful observation.
- ◆ Please notify your doctor if you have a chronic respiratory condition such as asthma, severe muscle weakness, muscle atrophy, or glaucoma.
- ◆ The effects of the medication might appear at a site that is different from the Botox injection site. Botox injected at a non-cervical (neck) location might result in difficulty in swallowing food or aspiration pneumonia from accidentally inhaling food. Extra caution is necessary for patients who already have difficulty swallowing food and pediatric patients with a severe disability.
- ◆ On rare occasions, heart-related symptoms, such as arrhythmia or myocardial infarction, might appear after treatment. The association between these symptoms and Botox is unknown. However, if you experience these symptoms, please contact your doctor.
- ◆ Please use caution when operating machinery, such as driving a car, because side effects such as the feeling of weakness, muscle weakness, dizziness and decreased visual acuity might appear after treatment.
- ◆ Patients who had been restricting their daily lives before treatment should avoid labor involving excessive muscle contraction after treatment with this medication and should gradually reintroduce activities.
- ◆ After treatment, the likelihood of falling is greater in patients with arm or leg spasms because of increased activity and changes in muscle strength and balance due to Botox treatment.
- ◆ Safety of the treatment has not been established in children younger than 15 years old for indications other than ≥2-year-old pediatric patients with cerebral palsy presenting talipes equinus caused by leg spasm, and ≥12-year-old strabismus patients. Death in pediatric patients during treatment with this medication has been reported overseas. Some of these patients had risk factors such as severe neuromuscular disease, difficulty swallowing, aspiration pneumonia, convulsions and heart disease. Careful observation is necessary when a pediatric patient with severe disability, such as patients with quadriplegia, those receiving enteral nutrition, or patients who have experienced aspiration pneumonia or lung disease in the past, receives treatment with this medication.

◆ If you are receiving botulinum toxin at another medical facility, you must inform your doctor about the date and dose of administration and the target disease for treatment.

Your personal information (such as initials, birth date and name of condition) will be provided to the manufacturer of this drug (GlaxoSmithKline, a third-party) for the purpose of maintaining proper use and compliance.

Consent form for treatment with Botox

I have received an explanation from a physician on the cautionary points concerning treatment with Botox for “spasmodic torticollis,” “arm spasm,” “leg spasm,” and “talipes equinus caused by leg spasm in ≥2-year-old pediatric patients with cerebral palsy.” I fully understand the explanation and agree to receive treatment with Botox.

Date of consent: month/day/year

[Patient] Address:

(telephone)

Name:

Birth date: month/day/year

[Patient representative] Address: (telephone)

Name:
Patient name:
Relationship to patient:
Date of explanation: month/day/year

[Date of explanation] month/day/year
Hospital name:
Department name:
Physician name:

○ You will not be penalized in the medical care you receive if you decline treatment with this medication before or during treatment.
