# Supplementary material for: Selection process for botulinum toxin injections in patients with chronic-stage hemiplegic stroke: a qualitative study
Source: BMC Med Inform Decis Mak. 2019 Dec 19;19:280. doi: 10.1186/s12911-019-1003-9 (PMC6923967; doi:10.1186/s12911-019-1003-9)
Supplement: Supplementary file 2 — Additional file 2. Table of contents of the booklet. The booklet was provided by a pharmaceutical company to be distributed to patients. We confirmed with the pharmaceutical company that created the booklet that they were only available in Japanese. Therefore, we translated the table of contents of the booklet from Japanese into English. [file 12911_2019_1003_MOESM2_ESM.pdf]

Additional file 2 table of contents of the booklet (The booklet was provided by GSK to be distributed to patients.)

## Table of Contents


### 1. Stroke

What is a stroke (a cerebrovascular disorder)?

Number of patients with stroke

Physical impairment with stroke

Rehabilitation in patients with stroke

### 2. Spasms

What is a spasm (tightening of hands and feet)?

Treatment of spasms (tightening of hands and feet)

### 3. Botulinum therapy

What is botulinum therapy?

Effects of botulinum therapy

Steps in botulinum therapy

Injection site

Side effects of botulinum therapy

Cautionary point 1: Before receiving botulinum therapy

Cautionary point 2: After receiving botulinum therapy

Botulinum therapy: Q&A

### 4. Stroke and public support

Physical disability certificate

High-cost medical expense benefit system

Long-term care insurance
